# Higher levels of unmet support needs in spouses are associated with poorer quality of life – a descriptive cross-sectional study in the context of palliative home care

**DOI:** 10.1186/s12904-021-00829-9

**Published:** 2021-08-28

**Authors:** Maria Norinder, Kristofer Årestedt, Susanne Lind, Lena Axelsson, Gunn Grande, Gail Ewing, Maja Holm, Joakim Öhlén, Inger Benkel, Anette Alvariza

**Affiliations:** 1 Department of Health Care Sciences, Palliative Research Centre, Ersta Sköndal Bräcke University College, Box 11189, 100 61 Stockholm, Sweden; 2 Capio Palliative Care, Dalen Hospital, 121 87 Stockholm, Sweden; 3 Faculty of Health and Life Sciences, Linnaeus University, 39182 Kalmar, Sweden; 4 The Research Section, Region Kalmar County, Kalmar, Sweden; 5 Department of Nursing Science, Sophiahemmet University, Stockholm, Sweden; 6 Division of Nursing, Midwifery & Social Care, Faculty of Biology, Medicine and Health, University of Manchester, Manchester, UK; 7 Centre for Family Research, University of Cambridge, Cambridge, UK; 8 Institute of Health and Care Sciences and the Centre for Person-Centred Care, Sahlgrenska Academy, University of Gothenburg, Gothenburg, Sweden; 9 The Palliative Care Unit, Sahlgrenska University Hospital, Gothenburg, Sweden; 10 Department of Geriatric Medicine, Sahlgrenska Academy, University of Gothenburg, Gothenburg, Sweden

**Keywords:** Family caregivers, Life-threatening illness, Palliative care, Quality of life, Support needs

## Abstract

**Background:**

Family caregivers often report having unmet support needs when caring for someone with life-threatening illness. They are at risk for psychological distress, adverse physical symptoms and negatively affected quality of life. This study aims to explore associations between family caregivers’ support needs and quality of life when caring for a spouse receiving specialized palliative home care.

**Methods:**

A descriptive cross-sectional design was used: 114 family caregivers completed the Carer Support Needs Assessment Tool (CSNAT) and the Quality of Life in Life-Threatening Illness – Family caregiver version (QOLLTI-F) and 43 of them also answered one open-ended question on thoughts about their situation. Descriptive statistics, multiple linear regression analyses, and qualitative content analysis, were used for analyses.

**Results:**

Higher levels of unmet support needs were significantly associated with poorer quality of life. All CSNAT support domains were significantly associated with one or more quality of life domains in QOLLTI-F, with the exception of the QoL domain related to distress about the patient condition. However, family caregivers described in the open-ended question that their life was disrupted by the patient’s life-threatening illness and its consequences. Family caregivers reported most the need of more support concerning knowing what to expect in the future, which they also described as worries and concerns about what the illness would mean for them and the patient further on. Lowest QoL was reported in relation to the patient’s condition, and the family caregiver’s own physical and emotional health.

**Conclusion:**

With a deeper understanding of the complexities of supporting family caregivers in palliative care, healthcare professionals might help to increase family caregivers’ QoL by revealing their problems and concerns. Thus, tailored support is needed.

## Background

Palliative care aims to support the quality of life (QoL) of both patients and family caregivers [1, 2]. Family caregivers, for example, spouses, children, parents or others who have a significant relationship with a person with life-threatening illness, are essential [3, 4], as many patients are cared for at home towards the end of life [5–7]. Although palliative home care is provided by professionals [8], family caregivers are crucial providers of social support [9, 10] and a great deal of caregiving [10, 11] involving practical, emotional and existential support [10–13]. Many report unmet support needs and insufficient knowledge of caregiving wanting more information about the illness’ prognosis, progression and treatment and emotional support for themselves [10, 14–16]. Support needs can change during the illness trajectory and unmet needs may negatively affect family caregivers’ QoL [17, 18].

Many family caregivers put their own lives on hold and attend to the patient’s needs. In addition, they must cope with the patient’s impending death and an uncertain future [13]. When confronted with life-threatening illness, existential concerns are often evoked, forcing family caregivers to confront life’s fragility and their own mortality [19]. Many exhibit feelings of helplessness and lack of control that could lead to anxiety, but also physical symptoms, such as fatigue and sleep deprivation [13]. Moreover, family caregivers often have higher levels of anxiety and depression than those reported in the general population [20]. Spouses are often the primary caregiver, providing more care and support which also tends to increase with age. They often report higher levels of distress and more physical and psychological burden [21]. Caregiver burden has been found to negatively affect family caregivers’ QoL. Adequate support might contribute to easing burden and thus improve QoL [22]. QoL is often negatively affected, both during caregiving and after the patient’s death [20, 23] and seems to decrease as the patient deteriorates. Their situation is interwoven with that of the patient [24, 25] and it can take several months after the patient’s death for their QoL to return to a normative standard [23]. QoL is an essential part of palliative care [26]. However, it is not always easy for healthcare professionals to adequately support the maintenance of family caregivers’ QoL, as it is a broad concept affected by a person’s physical and psychological health, social relationships and personal beliefs [2].

Existing literature contributes with knowledge concerning the need for support among family caregivers [27] and some studies indicate that caregiving has a significant impact on family caregivers [28]. However, studies often include small samples, using different family caregiver populations and have not looked at support needs in relation to separate domain of QoL. More knowledge is needed to better understand what may be helpful during the caring phase and the relation between separate domains of support needs and overall as well as various domains of QoL. It is also of importance to identify which domains of support needs that might be of particular significance for the QoL of family caregivers. Therefore, the aim of this study was to explore associations between family caregivers’ support needs and quality of life when caring for a spouse receiving specialized palliative home care.

## Method

### Design

This study has a descriptive cross-sectional design using both quantitative and qualitative data. The study was approved by a regional ethics review board in Sweden (No. 2015/1517–31/5).

### Study context and inclusion criteria

Data were collected at two specialized palliative home care services, each of which provided care for patients with life-threatening illness and palliative care needs, e.g., symptom management, and emotional and existential support, in two larger cities in different parts of Sweden. Both services were staffed by intra-professional teams (nurses, physicians, social workers, physical and occupational therapists). Inclusion criteria were: spouse or partner to and living with a person who received specialized palliative home care at one of the two included services; 18 years or older; able to read and understand Swedish. In the Swedish healthcare system, general palliative care can be provided in most healthcare settings. Specialized palliative care is provided in hospices, specialized palliative in-patient wards and home care services. Hospital bed numbers are decreasing, and an increased number of patients are cared for in their homes [29]. A social insurance system ensures that family caregivers are provided with an allowance for a limited period from the government to care for a severely ill family member at home.

### Procedure and data collection

All data were collected during 2016. Access to patient records was granted from the director of each department, and head nurses were asked to identify one family caregiver for each patient. Eligible family caregivers (*n* = 342) were identified via healthcare professionals and were contacted by the researchers with a letter sent by post requesting their participation, along with information about the study, a study-specific questionnaire, and a pre-paid stamped envelope for its return. The letter contained the phone number and email addresses of two of the researchers to allow participants to ask questions and receive oral information about the study. Participants were informed that participation was voluntary, and that data would be kept confidential. Altogether, 114 family caregivers returned the questionnaire with a signed consent form (response rate 33%).

### The Questionnaire

The questionnaire included demographic questions and validated tools/instruments; the *Carer Support Needs Assessment Tool* (CSNAT) [30] and the *Quality of Life in Life-Threatening Illness – Family caregiver version* (QOLLTI-F) [31]. In addition, an open-ended question was included at the end of the questionnaire: “Do you have any thoughts about your situation, not covered in the questionnaire, that you want to share?”.

*The CSNAT* was developed in the UK based on interviews with family caregivers concerning their perspectives of key aspects of support needed while palliative care was provided at home. The resultant tool has 14 questions, based on broad domains covering practical, emotional, existential and social support, which are intended to capture a range of underlying support needs that are meaningful for family caregivers. The domains address family caregivers’ dual roles of being a provider of care (enabling support) and a person who is in need of support her/himself (direct support). There is an additional ‘anything else’ section enabling caregivers to add any support needs not covered by the existing domains. The tool has four response options about the need for more support, ranging from ‘no’ to ‘very much more’ [30]. For this study, the CSNAT was used as a ‘research tool’ to solely identify unmet support needs using the version that has been translated and validated among Swedish family caregivers [32].

*The QOLLTI-F* was developed in Canada based on interviews with family caregivers of patients with cancer, and focusing on what was experienced as important for their own QoL. The QOLLTI-F, version 2, includes a total of 17 items divided into 7 subscales assessing different domains: environment, patient condition, the family caregiver’s own state, family caregiver’s outlook, quality of care, relationships and financial worries. It also includes 1 item about overall QoL. All items are scored on an 11-point numeric rating scale, ranging between 0–10 with a descriptive anchor at each extreme. Each subscale is calculated by adding the responses and dividing the sum by the number of items in each domain. Thus, each subscale can range between 0–10, and after reversed items have been rescored, higher scores indicate higher levels of QoL [31]. The QOLLTI-F has been translated and validated among Swedish family caregivers [33]. The Cronbach’s alpha for the subscales that include more than one item were 0.58 for *Environment*, 0.87 for *Family caregiver’s own state*, 0.65 for *Family caregiver’s outlook*, 0.94 for *Quality of care*, and 0.76 for *Relationships*.

### Data analysis

Descriptive statistics were used to present the characteristics of the family caregivers and the study variables. A series of multiple linear regression analyses were used to explore associations between support needs and QoL. The QOLLTI-F scales were used as outcome variables while the CSNAT items were used as explanatory variables. As the CSNAT response scale have one category that implies no support need while the other categories reflect various levels of support need, the CSNAT items were dichotomized into *‘No support need’* (= 0) and *‘Support need’* (= 1). The regression models were adjusted for sex (female = 0; male = 1), age, and education no university degree (= 0; university degree = 1). For all tests, *p* < 0.05 was considered to be statistically significant. All analyses were conducted in Stata 17.0 (StataCorp LLC, College Station, TX, USA).

The open-ended question was analysed with content analysis [34]. A total of 43 family caregivers (29 women and 14 men) provided comments that varied in length from a few lines to two extra pages. Many shared detailed, personal emotional experiences about their current situation, while others had written less, occasionally using strong expressions or words to share their views. In the initial reading of the comments it was found that they contained stories about their support needs and/or QoL. Next step was to read the text thoroughly to specifically search for various expressions. Associations between support needs and QoL were searched using the CSNAT and QOLLTI-F to help in identifying expressions concerning support needs and QoL. Data were coded and grouped into categories that consisted of descriptions illustrating the associations.

## Results

### Family caregiver characteristics

The sample consisted of 114 family caregivers with a mean age of 67.5 (SD = 10.9) years. Most participants were women (*n* = 69, 61%). In general, participants were well educated, with 42% (*n* = 47) having a university degree. The majority (*n* = 68, 61%) were retired, and about one-third (*n* = 32, 29%) were employed. A minority (*n* = 13, 12%) had children in the household. One-fifth (*n* = 22, 20%) reported receiving care benefits, i.e., they were paid by the Swedish government to provide care for the patient at home. Most of the patients had a cancer diagnosis (*n* = 96, 84%) or a cardiopulmonary disease (*n* = 15, 13%) (Table 1).

**Table 1:** Family caregiver characteristics (*n* = 114)

	Valid data, *n*	Descriptive statistics
Age (years), mean (SD) [min–max]	112	67.5 (10.9) [33–90]
Female sex, *n* (%)	114	69 (60.5)
Children in the household, *n* (%)	111	13 (11.7)
University education, *n* (%)	111	47 (42.3)
Occupation, *n* (%)	112	
Employed		32 (28.6)
Retired		68 (60.7)
Other		12 (10.7)
Benefit for care, *n* (%)	112	22 (19.6)
Patient characteristics		
Age (years), mean (SD) [min–max]	114	69.8 (12.1) [33–94]
Female sex, *n* (%)	114	44 (38.6)
Diagnosis, *n* (%)	114	
Cancer		96 (84.2)
Cardiopulmonary disease		15 (13.2)
Other		3 (2.6)

### Family caregivers’ support needs and quality of life

**Fig. 1:**
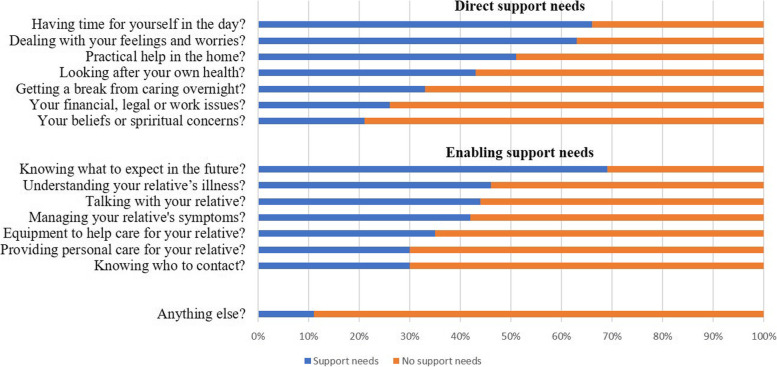
Family caregivers’ support needs assessed by CSNAT

The four domains where more than 50% of the family caregivers reported a need for more support were: *Knowing what to expect in the future* (69%), *Having time for oneself in the day* (66%), *Dealing with feelings and worries* (63%) and *Practical help in the home* (51%). Family caregivers were least likely to report the need for more support with *Beliefs or spiritual concerns* (21%) (Fig. 1).

**Fig. 2:**
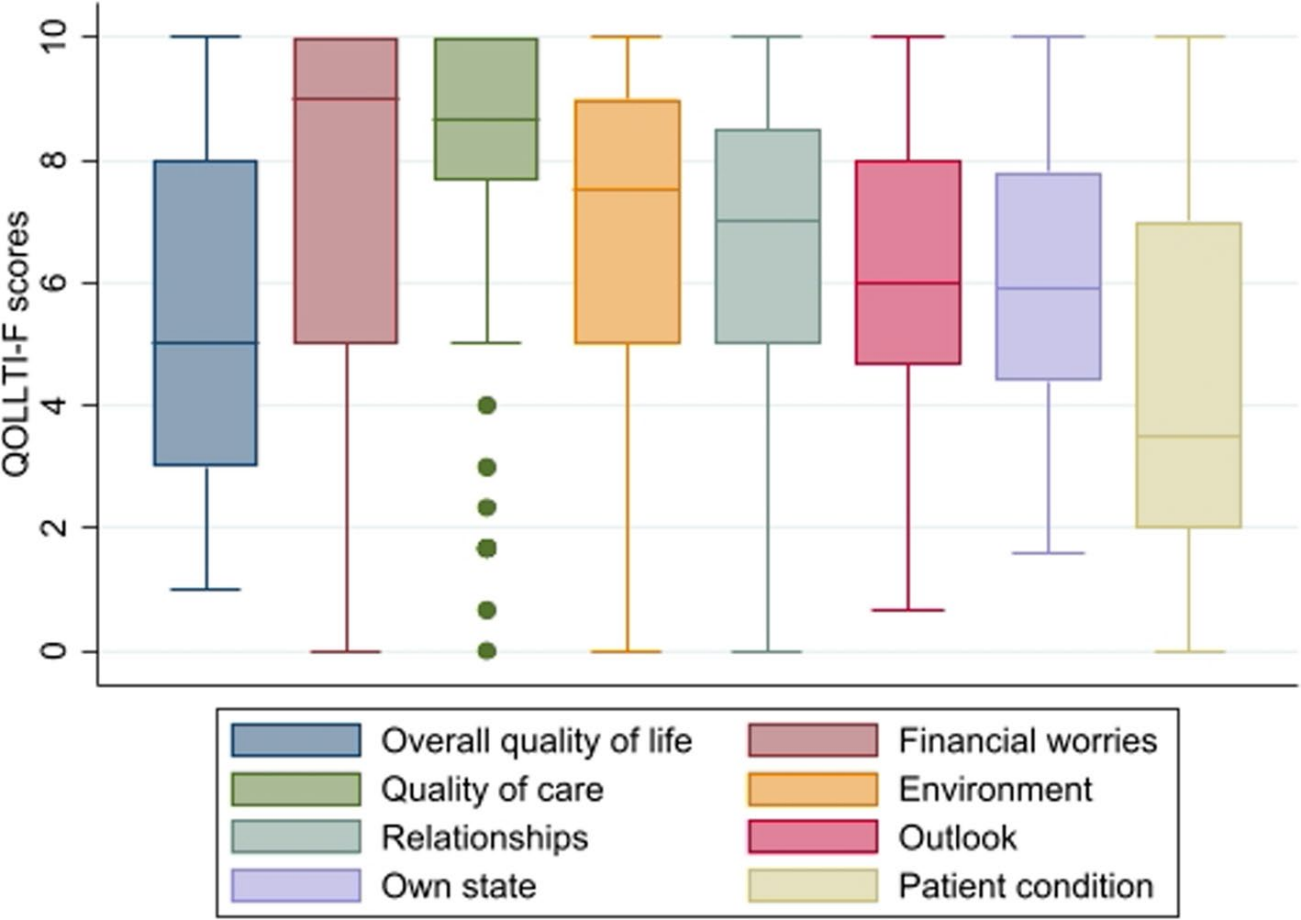
Box-plot of the quality of life domains meassured by the QOLLTI-F

The domain where family caregivers reported the poorest QoL was about *Patient condition* (Mdn = 3.5, q1–q3 = 1.75–7), followed by *Family caregiver’s own state* (Mdn = 6, q1–q3 = 4.6–8) and *Family caregiver’s outlook* (Mdn = 6, q1–q3 = 4.6–8). They reported on average the highest level of QoL in the domain *Financial worries* (Mdn = 9, q1–q3 = 5–10) and *Quality of care* (Mdn = 8.6, q1–q3 = 7.6–10). The median score on the domain *Overall quality of life* was 5 (q1–q3 = 3–8) (Table 2 and Fig. 2).

**Table 2:** Quality of life among family caregivers based on the Quality of Life in Life Threatening Illness – Family caregiver version (QOLLTI-F)

Description of item content	Domain	Mdn (q1–q3)
Overall QoL	Overall QoL	5.0 (3–8)
Satisfaction with place of care	Environment	7.5 (5–8.5)
Privacy		
Distress related to patient condition	Patient condition	3.5 (1.75–7)
Control over life	Family caregiver’s own state	6.0 (4.6–8)
Time to take care of oneself		
Clarity of thought		
Physical state		
Emotional state		
Feeling about caring for the family member (patient)	Family caregiver’s outlook	6.0 (4.6–8)
Comfort from outlook, faith or spirituality		
Meaning in life		
Agreement with decision making process for patient	Quality of care	8.6 (7.6–10)
Availability of health care		
Quality of care		
Interaction with patient	Relationships	7.5 (5–8.5)
Interaction with the other important people		
Stress due to financial situation	Financial worries	9.0 (5–10)

### Associations between domains of support needs and domains and overall quality of life

In general, higher levels of need for more support were significantly associated with poorer QoL. All of the domains of support needs were significantly associated with one or more QoL domains; in particular: *practical help in the home* (CSNAT 12, *B* = -1.31 to -2.33), *dealing with feelings and worries* (CSNAT 6, *B* = -1.17 to -1.51), *talking with your relative about his or her illness* (CSNAT 11, *B* = -1.03 to -1.82)*, having time for yourself during the day* (CSNAT 2, *B* = -1.25 to -1.88), *equipment to help care for your relative (*CSNAT 9, *B* = -0.98 to 1.87*)*, and *your beliefs or spiritual concerns* (CSNAT 10, *B* = -1.10 to -2.65). None of the domains of support needs were significantly associated with the QoL domain *Patient condition*, while 11 of 14 were associated with the domain *Family caregiver’s own state* (*B* = -0.93 to -1.59) (Table 3).

**Table 3:** Associations between domains of support needs measured by CSNAT and quality of life domains measured by QOLLTI-F based on multiple linear regression adjusted for sex, age and education

	Overall quality of life B	Environment B	Patient condition B	Family caregiver’s own state B	Family caregiver’s outlook B	Relationships B	Quality of care B	Financial worries B
CSNAT 1 Understanding your relative’s illness?	-0.69	-0.27	-0.71	-0.72	-0.82	-0.25	-1.43***	-0.19
CSNAT 2 Having time for yourself in the day?	-1.88***	-1.86***	-0.59	-1.59***	-1.25*	-1.64***	-0.44	-1.27
CSNAT 3 Managing your relative’s symptoms, including giving medicines?	-0.95*	-0.18	-0.18	-0.57	-0.34	-0.30	-0.82	-1.77*
CSNAT 4 Your financial, legal or work issues?	-0.83	-0.76	-0.62	-1.02*	-0.91	-0.93	-0.81	-3.44***
CSNAT 5 Providing personal care for your relative (eg dressing, washing, toileting)?	-1.14*	-0.97*	-0.67	-1.10**	-0.59	-0.43	-0.94	-1.73**
CSNAT 6 Dealing with your feelings and worries?	-1.51**	-1.20**	-0.96	-1.40***	-1.17*	-1.34**	-1.32**	-1.47*
CSNAT 7 Knowing who to contact if you are concerned about your relative (for a range of needs including at night)?	-0.78	-0.96*	0.34	-0.93*	-0.68	-0.39	-1.85***	-1.67*
CSNAT 8 Looking after your own health (physical problems)?	-0.99*	-1.27**	-0.41	-1.00*	-0.80	-0.73	-0.89	-0.87
CSNAT 9 Equipment to help care for your relative?	-0.98*	-1.08*	0.23	-1.18*	-0.75	-0.83	1.87***	-1.53*
CSNAT 10 Your beliefs or spiritual concerns?	-1.40*	-1.10*	0.77	-1.47***	-0.91	0.19	-1.18*	-2.65***
CSNAT 11 Talking with your relative about his or her illness?	-1.57***	-1.03*	-0.68	-1.10**	-1.32**	-0.47	-1.82***	-1.52*
CSNAT 12 Practical help in the home?	-1.31**	-1.58***	-0.14	-1.47***	-1.34***	-1.56***	-1.50***	-2.33***
CSNAT 13 Knowing what to expect in the future when caring for your relative?	-0.55	-0.57	-0.32	-0.54	-1.05*	-0.99*	-1.36**	-1.62*
CSNAT 14 Getting a break from caring overnight	-1.39**	-1.03*	-0.80	-1.37***	-0.87	-0.51	-0.90	-1.63*

Linear regression analyses between each suport need domain and quality of life scale, controlled for age, sex and education

*CSNAT* Carer Support Needs Assessment Tool, *QOLLTI-F* The Quality of Life in Life-Threatening Illness – Family carer/caregiver version

*B* = *Unstandardized sloop coefficeint, p* < 0.05, **p < 0.01, ***p < 0.001

### Family caregivers’ comments – reflecting associations between support needs and quality of life

The analysis of comments revealed that, especially, the need for more support in *Looking after one’s own health*, *Dealing with feelings and worries* and *Having time for oneself* appeared to be associated with family caregivers’ QoL.

#### Own health and quality of life

Family caregivers commented that they needed more support with looking after their own health as they often prioritised the patient over themselves. They had to support the patient both emotionally and physically, resulting in neglecting their own health. Their situation, with limited sleep and increased care responsibilities at home, made them feel extremely tired, affecting their QoL in terms of both physical and emotional health. A 55-year-old woman wrote that she was on full-time sick leave while caring for her husband. She did not dare sleep at night and that clearly affected her own health. Some caregivers already had severe health problems of their own, which had worsened due to their situation. A 86-year-old woman wrote*: “My back pain and breathlessness had become much worse because of the care of my husband”.* Alternatively, those who wrote that they continued to look after their own health with, for example, recreational or sport activities, believed that it made them feel physically and mentally healthier. A 71- year-old man described that three times a week, he went to the gym and occasionally played in an orchestra. He prioritized these activities more than anything else.

#### Dealing with feelings and worries and quality of life

Many comments were about feelings and worries related to their current life and to the future.

Family caregivers feared that their support and care would not be sufficient to relieve the patient’s symptoms. One woman, aged 66 years, wrote: “*How much medicine do I dare to use and combine? What helps and what can be the opposite? When it lasts for several hours it takes time to dare to relax before the next round of pain comes”*. The comments revealed that they needed more support to deal with worries for the unknown future and how things would be if the situation did not improve. A 53-year-old woman reported that she was constantly worried about their uncertain situation and her ill husband, not knowing how long he would live and whether he would suffer from his illness. These kinds of worries were written by many, and the comments also concerned thoughts about how they would manage to be involuntarily alone. All these concerns clearly impacted their QoL.

#### Having time for oneself and quality of life

Family caregivers wrote about how they, due to their caregiving situation, needed support to find time for themselves during the day. Some could not leave the patient alone at home. Many had the main responsibility for care around the clock and those with small children wrote that help with taking care of the children would have been helpful. A 55-year-old woman described her situation: “*Absolutely no time for myself since I take care of the children at the same time. It goes on, without any break*”. This was also raised by a 50-year-old man: *“In my case I have a 1,5-year old child which makes the whole situation much more stressful”.* Family caregivers’ support needs in finding time for themselves was also related to their relationship with the patient. Their spousal relationship was already strained by the new caregiver relationship, and the lack of time for themselves contributed further to stressful interactions between them. Family caregivers also needed time for themselves to take care of other relationships that were important to them.

## Discussion

This study found that higher levels of support needs were significantly associated with poorer QoL in family caregivers who cared for a spouse receiving specialised palliative care at home. All of the support domains on CSNAT were significantly associated with one or more QoL domains in QOLLTI-F, with the exception of QoL related to distress about the patient’s condition. However, family caregivers described in the open-ended question that their life was disrupted by the patient’s life-threatening illness and it affected their QoL relating to both physical and emotional health.

Family caregivers in the present study reported most need of additional support with *Knowing what to expect in the future* and *Having time for yourself in the day*. This is in line with the results from a study using the CSNAT among Swedish family caregivers of patients going through allogenic stemcell transplantion [35] and family caregivers in United Kingdom [30], Australia [36] and China [37]. Lowest QoL was reported in the QOLLTI-F domains related to the *Patient condition, and Family caregivers’ own state.* This indicates a need for more knowledge about the disease and how it will potentially affect the patient’ and the family caregivers’ future. The lack of this knowledge in turn seems to affect their QoL.

The majority of the CSNAT domains where more support was needed were associated with lower QoL related to the family caregivers’ own state, as they prioritised the patient’s needs over their own. Palliative care philosophy and definition stresses that patients and their family caregivers should be seen as the unit of care [38]. While this should suggest the family caregiver is supported, this is not always the case. The family caregiver and the patient have different needs, yet the patient’s views in fact often take precedence over those of the family caregiver, thus resulting in a risk that family caregivers’ views or needs are not taken into account [39, 40]. In addition, many family caregivers are reluctant to express difficulties or disclose their own needs to healthcare professionals and to ask for help [41]. In the present study, several free-text comments were related to psychological burden, social isolation and reduced time caused by caregiving responsibilities. They stressed the need for respite care or some own free time. This discrepancy between the lack of support opportunities and the desire for more free time has the potential to increase the burden for family caregivers in need of more time off [42]. Allocation of support is also dependent on whether family caregivers clearly express their need for support [43]. This suggests that it is important for healthcare professionals to pay attention to, assess and support family caregivers with their own support needs to successfully promote their QoL.

The top four support needs reported by family caregivers were each associated with poorer QoL in most of the QoL domains. A recent study found all CSNAT domains to be associated with a negative impact of the overall QoL [37]. In the present study, support needs, where unmet support needs were within the “direct” support domains, family caregivers’ QoL was affected. This is in line with previous research that shows that family caregivers, in addition to information and educational needs, also need support from healthcare professionals to prioritise caring for their own health and well-being [44].

The present study found no association between family caregivers’ need for more support and their QoL related to distress concerning the *Patient condition*, even though almost half reported that they wanted more support with *Understanding their relative’s illness*. Consistent with the results of other studies [45, 46], many family caregivers in the present study commented that they were constantly worried about how the illness would affect them both in the future, whether the patient would suffer, and how they would be able to manage the symptoms. When the illness progresses to a more advanced stage and patient care becomes more complex, the family caregivers’ distress often increases [45, 47] as more demands are placed on them [48]. In addition, family caregivers often face further distress when witnessing the dying process, which is often accompanied by physical deterioration and the patient’s loss of dignity [13, 45], and the higher amount of time that family caregivers devote to the patient is associated with poorer QoL [49]. In line with this, family caregivers in the present study reported the poorest QoL within the domain related to concerns about the *Patient’s condition*, which is also highlighted in the comments. These findings are interesting in relation to the fact that the QOLLTI-F itself takes into account how the QoL of family caregivers is affected by the distress related to the patient’s condition [31]. Thus, in the present study, performed in a specialized palliative care context, one could see that the patient’s condition does affect the family caregiver, but it was not associated with their need for more support, even though the CSNAT identifies support needs to provide care for the patient. Consequently, it could be assumed that patients received high quality care with adequate symptom management provided by the specialized palliative homecare settings. In addition, it should be noted that the CSNAT tool specifically focuses on the family caregiver and what support he or she needs to provide care or to maintain their own well-being. However, the patient and the family caregiver’s situation are interwoven, and they are both faced with considerable stress from physical symptoms and psychosocial burdens. They use multiple forms of coping through the illness trajectory that can help them manage the disease and related symptoms [50]. The family caregivers in this study may already have learned how to use problem-focused strategies for active management of practical stressors but have more difficult to handle the emotional distress.

To better understand this study’s results, family caregivers’ support needs and QoL can be enhanced by using theory. Andershed and Ternestedt’s (1999; 2001) theoretical framework focuses on the involvement and principal needs of family caregivers in palliative care [51, 52]. When family caregivers feel confirmed, informed and well supported by healthcare professionals, it increases the possibility that better QoL for both patients and family caregivers can be promoted, and facilitates the conditions necessary for providing meaningful care. In order to achieve this, family caregivers need support according to the three key concepts; “Knowing” (informational needs), “Being” (existential and emotional needs) and “Doing” (practical needs). All three of these key concepts can be found in the reported support and QoL domains in this study. However, the relationship between the three key concepts and the support and QoL domains is not a simple direct relationship. “*Support with knowing what to expect in the future*” may, for instance, be about a need for information (knowing), but could also be about emotional support and existential concerns in terms of anxiety about an uncertain future (being), or about finding out what practical measures need to be put in place as the patient deteriorates (doing). Such is the broad nature of the domains that further information, and emotional or practical support may be required for any of the domains, depending on the underlying support needs that the caregiver expresses, which in turn will affect their QoL.

## Methodological considerations

This study has some limitations that need to be considered. First, the cross-sectional design did not allow for any conclusions about the causal relationships between the variables. Second, the study was based on questionnares with closed-ended questions; only one open-ended question to explore the family caregivers situation was included.

The internal consistency assessed by Cronbach’s alpha was low for the *Envoronment* and *Family caregiver’s outlook* subscales in the QOLLTI-F. This was partly expected since both scales consists of few items, which decresed the Cronbach’s alpha coeffiicient [53]. In addition, both subscales demontreted low internal consitency reliability in the development of the QOLLTI-F [54]. Also, the response rate was low and, as the ethics approval did not include asking caregivers to provide their reasons for declining, no information exists about whether those declining differed from those who participated; however, it might be due to the stressful situation of caregiving. The sample in this present study had a high educational level and most were born in Sweden. They also received economic benefits from the government and the treatments provided by the palliative homecare service were free of charge. It is a well-known fact that family caregivers who are coping well are more likely to take part in research than those who are more vulnerable, which also means that this study may not capture the full representation of family caregivers [55]. Despite this limitation, the chosen methods reveal that there are subtle and nuanced relationships between family caregivers’ needs for more support and their QoL that may be even more pronounced in those who are less likely to take part in research.

## Conclusions and clinical implications

This study, performed in specialised palliative home care, shows associations between family caregiver’s need for more support and their QoL. Higher levels of support needs were significantly associated with poorer QoL for family caregivers. This gives additional weight to the importance of addressing the family caregivers’ needs for support. In this research, CSNAT was used as a research tool. For use in practice, the CSNAT is a communication tool that is integrated into a person-centered process of assessment and support. The response categories can facilitate communication with opportunities to express individual problems and concerns enabling more tailored support to address family caregivers’ specific needs that can enable healthcare professionals to give individual support to them. With a deeper understanding of the complexities of supporting family caregivers in palliative care, healthcare professionals are better placed to increase family caregivers’ QoL.

## Data Availability

The datasets used and/or analysed during the current study are available from the corresponding author on reasonable request.

## Abbreviations

CSNAT: The Carer Support Needs Assessment Tool
QoL: Quality of life
QOLLTI-F: Quality of Life in Life-Threatening Illness – Family caregiver version
